# Enzymatic Behavior of Condoliase in Porcine Nucleus Pulposus: Ex Vivo and In Vitro Assessment

**DOI:** 10.1002/jsp2.70131

**Published:** 2025-10-19

**Authors:** Ippei Watanabe, Ko Takeda, Taiichi Shirogane

**Affiliations:** ^1^ Medical Affairs Seikagaku Corporation Tokyo Japan; ^2^ Central Research Laboratories Seikagaku Corporation Tokyo Japan

**Keywords:** chemonucleolysis, chondroitin sulfate, condoliase, lumbar disc herniation, nucleus pulposus

## Abstract

**Background:**

Condoliase is a treatment for lumbar disc herniation. This enzyme exerts its medicinal effects by digesting chondroitin sulfate (CS), which is abundant in the nucleus pulposus. However, the behavior of administered condoliase in the nucleus pulposus is not clear. Because the purpose of this study is to understand the mechanism of enzyme action, we evaluated the properties of condoliase in the nucleus pulposus.

**Methods:**

The following were evaluated: (1) The diffusibility of fluorescein labeled condoliase injected into isolated porcine nucleus pulposus. (2) The time dependence of condoliase activity in porcine nucleus pulposus or CS solution. (3) The morphology of the enzyme‐treated nucleus pulposus tissue was characterized using scanning electron microscopy.

**Results:**

After injection into the nucleus pulposus, condoliase was difficult to diffuse spontaneously, and the rate of CS‐disaccharides production was significantly increased up to a peak at 24 h and decreased thereafter. Not all of the CS in the nucleus pulposus was digested by condoliase. These results suggested that condoliase digested CS locally without causing its spontaneous diffusion within the nucleus pulposus. Moreover, condoliase did not digest the collagen fibers that form the supportive architecture of the nucleus pulposus.

**Conclusions:**

We demonstrated that condoliase is retained in the nucleus pulposus and exerts its pharmacological effects by locally degrading CS without degrading collagen fibers. The results obtained in this study can be useful in predicting the mechanism of the pharmacological action of condoliase in clinical practice.

## Graphical abstract

Condoliase digested chondroitin sulfate locally, and did not digest the collagen fibers that form the supportive architecture of the nucleus pulposus.

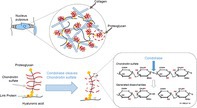

## 1 Introduction

Lumbar disc herniation (LDH) is caused by extrusion of the nucleus pulposus through the posterior annulus fibrosus into the spinal canal, leading to compression of the spinal nerve roots, causing back and leg pain. In the 1960s, chymopapain, a proteolytic enzyme, was used as an intermediate option between conservative and surgical treatment for LDH in Europe and the United States [1]. Since 2018, condoliase, a chondroitin sulfate (CS) degrading enzyme, has been used as a treatment for LDH in Japan [2, 3, 4]. Condoliase specifically digests the CS that abundantly presents in the nucleus pulposus, thereby reducing the pressure in the intervertebral disc (IVD) and on the nerve root. Currently, a phase III trial has been completed in the United States [5].

Condoliase cleaves the glycosidic bond between D‐glucuronic acid (GlcA) and *N*‐acetyl galactosamine (GalNAc) in the CS molecule in an elimination‐reactive manner, and in the process, generates a double bond between C4 and C5 at the nonreducing end of GlcA (Δ4,5GlcA). As a result, the unsaturated disaccharide [ΔDi‐xS: D4,5GlcA‐GalNAc (xS)] is the final degradation product [6, 7]. CS mainly exists in the following isomeric forms depending on the binding position and number of sulfate groups attached to GlcA or GalNAc: non‐sulfated chondroitin (CH), chondroitin 4‐sulfate (CSA), and chondroitin 6‐sulfate (CSC). The main disaccharide is the A‐unit [GlcAβ1‐3GalNAc(4S)] in CSA and the C‐unit [GlcAβ1‐3GalNAc(6S)] in CSC. The CS in human nucleus pulposus is mostly composed of A‐ and C‐units, with the latter accounting for more than 80% of the composition ratio [8]. Since condoliase shows high reactivity toward CSA and CSC, it can be said to be an effective drug against LDH [9, 10].

The pharmacological effects of condoliase have been evaluated [11, 12], but the details of drug diffusibility and activity in the nucleus pulposus after condoliase administration are unknown. Condoliase was anticipated to have less adverse effect on the IVD tissue due to the lack of proteolytic activity. However, no reports have evaluated the morphology of the nucleus pulposus after the administration of condoliase using scanning electron microscopy (SEM). In this study, we evaluate the enzymatic behavior of condoliase in the nucleus pulposus and the morphology of the tissues after condoliase treatment in isolated porcine nucleus pulposus tissues. The findings obtained in this study may provide useful information to understand the properties of condoliase used to treat LDH.

## 2 Materials and Methods

### 2.1 Materials

CSA (derived from whale cartilage) was purchased from Carbosynth Ltd. (Berks, UK). CSC (derived from shark cartilage) was obtained from Seikagaku Corp. (Tokyo, Japan). The average molecular weight (MW) of CSA or CSC measured according to the following method was 20.4 and 45.6 kDa, respectively. Purified condoliase derived from *P. vulgaris* NCTC 4636 was obtained from Seikagaku Corp. Purified collagenase derived from *Clostridium histolyticum was purchased from* Nordmark Pharma GmbH (Uetersen, Germany). Actinase E from *Streptomyces griseus* was purchased from Kaken Pharmaceutical Co Ltd. (Tokyo, Japan). Fluorescite was purchased from Novartis International AG (Basel, Switzerland). Porcine backbone was purchased from Tokyo Shibaurazoki Corp. (Tokyo, Japan). The backbone was harvested from 6‐month‐old females or castrated males, which were hybrids of four breeds: Landrace, Yorkshire, Berkshire, and Duroc. The porcine nucleus pulposus harvested from backbone was used in this study.

### 2.2 Analysis of MW and Disaccharide Composition of CS

The MW and disaccharide composition of CS were determined according to the method of Watanabe et al. [13]. The MW of CS was estimated by a high‐performance size‐exclusion chromatography (HPSEC) system (Shimazu Corp., Kyoto, Japan) equipped with gel exclusion columns, TSK‐gel PWXLG4000, 3000, and 2500 (Tosoh Corp., Tokyo, Japan). MW was calculated by LC solution software (Tosoh Corp.) using a CS of known absolute MW as a standard. Each unsaturated CS‐disaccharide was quantified using a high‐performance liquid chromatography (HPLC) system equipped with a reverse phase column, DOCOSIL SP 100 column (Senshu Scientific Co. Ltd., Tokyo, Japan).

### 2.3 Time Course of Condoliase Digestion of CS

One milliliter of CSA (1 mg/mL) in 50 mM Tris–HCl buffer (pH 7.0) was mixed with 0.1 mU of condoliase, and the mixture was incubated at 37°C for 14 days. According to the HPLC method described above, profiles of digestion products and generated unsaturated CS‐disaccharides (nmol/mL) digested with condoliase in the reaction solution were evaluated every other day (*n* = 3). The trend of CS‐disaccharides production over time was analyzed by stepwise linear regression, with the breakpoint at the 10th day after the start of the reaction. *p* values of less than 0.05 were considered significant.

### 2.4 Analysis of CS Contained in Porcine Nucleus Pulposus

Exactly 0.5 g of harvested porcine nucleus pulposus was freeze‐dried overnight. The nucleus pulposus after drying weighed 85 mg. The freeze‐dried sample was immersed in 2% actinase solution (in 10 mM Tris–HCl buffer pH 8.0) and incubated at 55°C for 16 h or more. The suspension was treated at 100°C for 10 min and filtered through a 0.22 μm filter. The MW of CS contained in the solution was measured according to the method described above. Furthermore, 2 U condoliase was added to 1 mL of the solution, and the mixture was incubated overnight at 37°C. As a control, 1 mL of CSC (1 mg/mL) was digested with 0.25 U condoliase. The profiles of digestion products and the disaccharide composition of CS were analyzed using the method described above.

### 2.5 Preparation of Fluorescein Labeled Condoliase

Fluorescein labeled condoliase (F‐condoliase) was prepared using the Fluorescein Labeling Kit‐NH_2_ (Dojindo Laboratories, Kumamoto, Japan). The preparation method followed the product instructions. The side chains of lysine residues and the NH_2_ group of the N‐terminal of condoliase were labeled with fluorescein. To evaluate the activity of F‐condoliase, 1 mL of CSC (1 mg/mL) in 50 mM Tris–HCl buffer (pH 7.0) was mixed with 10 μL of F‐condoliase and incubated at 37°C for 3 h (*n* = 3). During incubation, the time‐dependent absorbance at 232 nm of the mixture was measured using a spectrophotometer UV‐1900i (Shimazu Corporation, Kyoto Japan). To estimate the labeling rate (number of fluorescein molecules per protein molecule), the absorbance of *F‐condoliase* was measured at 280 nm and 500 nm (A_280_ and A_500_) using a spectrophotometer. The labeling rate was calculated by using the following formula: A_500_/molar extinction coefficient of fluorescein/A_280_‐A_500_ × 0.22/molar extinction coefficient for condoliase at 280 nm. The molar extinction coefficient of fluorescein is 60,000 L mol^−1^ cm^−1^. The molar extinction coefficient for condoliase at 280 nm (L mol^−1^ cm^−1^) was calculated by using the following formula: A_280_/condoliase concentration (mol/L) × path length of a cuvette (1 cm).

### 2.6 Behavior of F‐Condoliase in the Nucleus Pulposus

Fluorescite was diluted with saline to prepare a 25 μg/mL fluorescein solution. Approximately 0.1 g of excised nucleus pulposus tissue was injected with 10–12 μL of F‐condoliase or fluorescein (*n* = 3 or 4). The nucleus pulposus tissue was wrapped in plastic wrap to prevent drying, placed in a Petri dish, and stored in an incubator at 37°C for 3 h. The behavior of the injected drug was observed over time with a Leica THUNDER Imager 3D Tissue (Leica Microsystems, Wetzlar, Germany) using a LAS‐X Navigator software module with a 5× objective lens. To measure the area of F‐condoliase or fluorescein injected in the nucleus pulposus, the images taken were analyzed using ImageJ software. The procedure is briefly described in Figure S1. To set the threshold for the fluorescent signal, the lower and upper threshold values for binarization were set at 23 and 100, respectively. The area of the fluorescent signal (mm^2^) was selected automatically and calculated.

### 2.7 Enzyme Activity of Condoliase in Nucleus Pulposus

Nucleus pulposus (each weighing 0.1 g) was injected with 12 μL of 1.25 U/mL (a total of 15 mU per tissue) condoliase (dissolved in saline), incubated at 37°C for 1, 3, 6, 12, 24, or 48 h, and then heated at 100°C for 1 min. After freeze‐drying, each nucleus pulposus sample was immersed in 2% actinase solution (in 10 mM Tris–HCl buffer pH 8.0) and incubated at 55°C for 16 h or more. The suspension was treated at 100°C for 10 min and filtered through a 0.22 μm filter. CS unsaturated disaccharides, ΔDi‐0S, ΔDi‐4S, and ΔDi‐6S (derived from 0S‐unit, A‐unit, and C‐unit, respectively), were quantified according to the method described above (*n* = 3 or 4). The total amount of ΔDi‐0S, ΔDi‐4S, and ΔDi‐6S, which are the main components of CS in the nucleus pulposus, was calculated as generated disaccharides (nmol) and divided by the weight of dry tissue (mg). Enzyme activity was evaluated based on CS unsaturated disaccharides produced over time. The trend of CS‐disaccharides production over time was analyzed by stepwise linear regression with the breakpoint set at 24 h after the start of the reaction. *p* values of less than 0.05 were considered significant.

### 2.8 SEM Images of Enzyme‐Treated Nucleus Pulposus

Approximately 0.1 g of excised porcine nucleus pulposus was immersed in saline, 1.25 U condoliase (dissolved in saline), or 1 U collagenase (dissolved in saline), and incubated at 37°C.

Incubation times were 6, 12, and 24 h with saline and condoliase, and 1, 12, and 24 h with collagenase (*n* = 3). In this study, collagenase was used as a research reagent to identify collagen, not for the purpose of evaluating its properties. After incubation, a small fragment of the nucleus pulposus was treated as follows: (i) primary fixation with 2.5% glutaraldehyde, (ii) secondary fixation with 1.5% osmium tetroxide, (iii) ethanol‐dehydration, (iv) critical‐point drying, and (v) osmium plasma coating. The morphology of the tissue treated by the above steps was characterized using the JSM‐7900F scanning electron microscope (JEOL Ltd., Tokyo, Japan). Each tissue was observed at a magnification range from ×1000 to ×10 000. The acceleration voltage was set to 1 kV.

## 3 Results

### 3.1 Properties of CS Contained in Porcine Nucleus Pulposus

**FIGURE 1:**
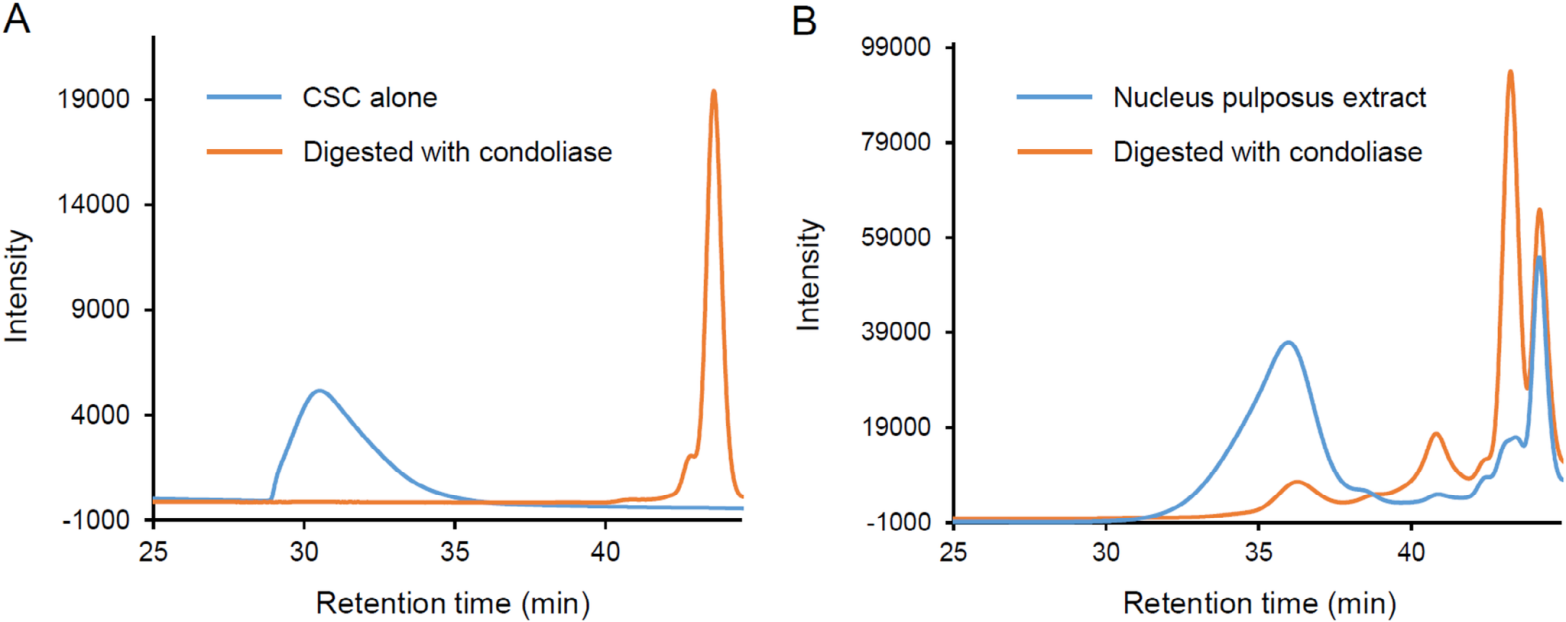
HPSEC profiles of CS and condoliase digestion products. (A) HPSEC profiles of undigested CS derived from shark and the digestion products of CS. One mL of CSC (1 mg/mL) was digested overnight with condoliase (0.25 U) at pH 8.0 and 37°C. (B) HPSEC profiles of extract solution from porcine nucleus pulposus and the digestion products. One mL of extract solution from porcine nucleus pulposus was digested overnight with condoliase (2 U) at pH 8.0 and 37°C.

The retention time (RT) of CS (derived from shark) was observed at 30 min. When adding 0.25 U of condoliase, the peak at RT 28–35 min disappeared completely, and CS unsaturated disaccharides (RT: 43 min) were produced (Figure 1A). CS with a MW of 45.6 kDa was completely degraded under the enzyme treatment. In the profile of the porcine nucleus pulposus sample, the peak at RT 35 min almost disappeared after treatment with condoliase, and disaccharides were produced (Figure 1B). Therefore, the peak at RT 31–38 min was CS derived from nucleus pulposus, and the MW of CS was 7.6 kDa (Table 1). In addition, similar to the extraction of CS from porcine nucleus pulposus, when CS derived from shark was treated with actinase or boiling, the MW of CS did not decrease (Figure S2). The disaccharide composition of CS derived from porcine nucleus pulposus was as follows: ΔDi‐0S; 4.0%, ΔDi‐4S; 11.3%, ΔDi‐6S; 82.9%, ΔDi‐4,6S; 1.0%, and ΔDi‐2,6S; 0.7%. The total amount of CS disaccharide in a 1 mg freeze‐dried sample was 740 nmol, and the breakdown of the amount of each CS disaccharide isomer (nmol/mg of dry tissue) was as follows: ΔDi‐0S; 29.9, ΔDi‐4S; 83.8, ΔDi‐6S; 613.4, ΔDi‐4,6S; 7.6, and ΔDi‐2,6S; 5.2.

**TABLE 1:** MW and disaccharide compositions of CS derived from porcine nucleus pulposus.

CS	Mw (kDa)	Disaccharide composition (%)				
		ΔDi‐				
		0S	4S	6S	(4,6)S	(2,6)S
	7.6	4.0	11.3	82.9	1.0	0.7

### 3.2 Diffusibility of F‐Condoliase in the Nucleus Pulposus

**FIGURE 2:**
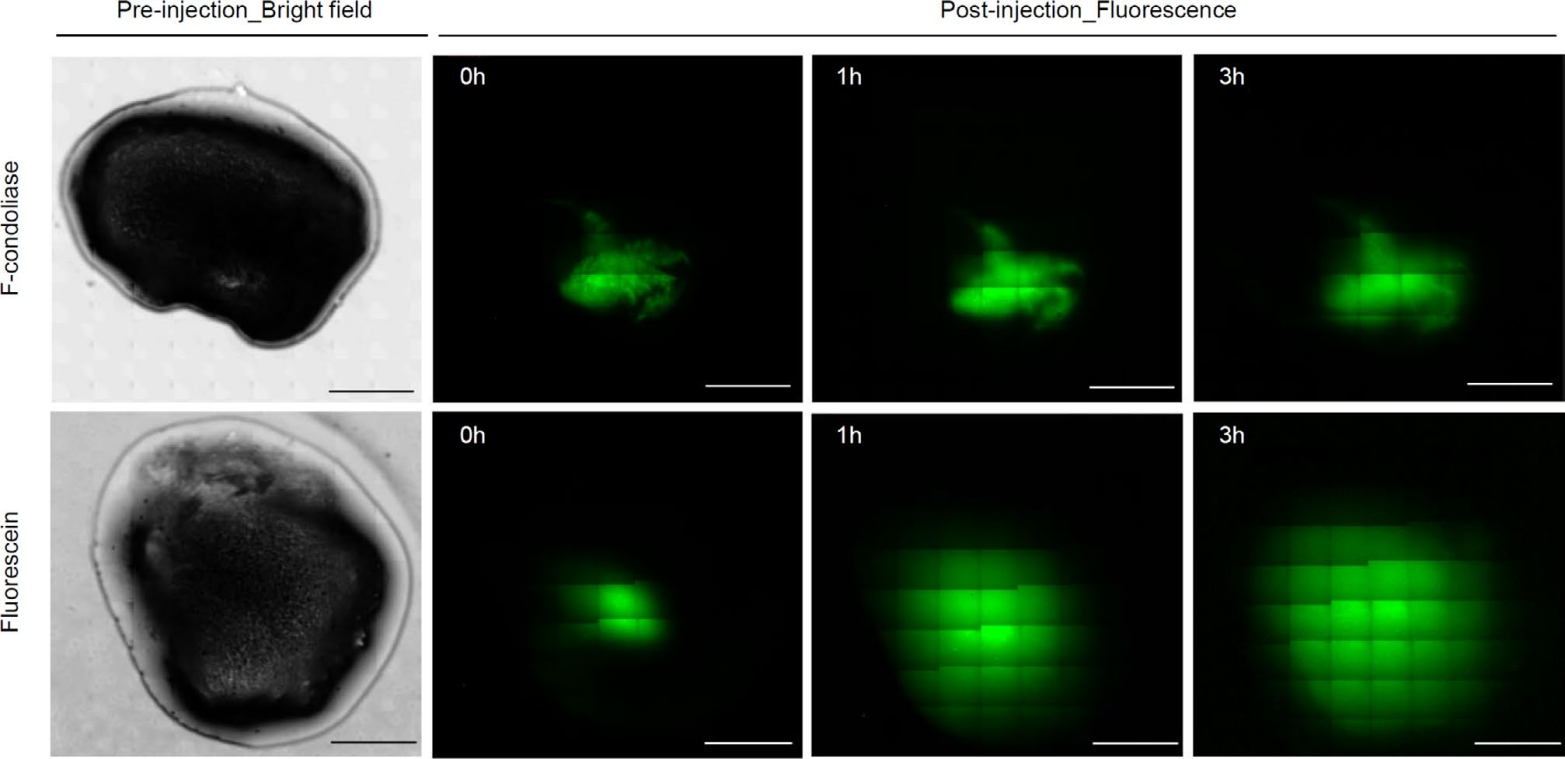
Time course of the spreadability of F‐condoliase in the nucleus pulposus. Bright field and fluorescence images of F‐condoliase (upper) or fluorescein (lower) in the excised porcine nucleus pulposus. Excised nucleus pulposus tissues were injected with 10–12 μL (300–360 mU) of F‐condoliase or fluorescein and incubated at 37°C for 3 h. At 0, 1, and 3 h after injection, each tissue was observed. Scale bar = 5 mm.

The molar extinction coefficient of condoliase at 280 nm was 3 × 10^5^. The ratio 280 nm / 500 nm of F‐condoliase was 1.6 (Figure S3), and the labeling rate was 3.5. In other words, about 3 molecules of fluorescein were bound to one molecule of condoliase. Labeling with fluorescein did not abolish enzyme activity, as F‐condoliase continued to cleave CS for at least 3 h (Figure S4). The prepared F‐condoliase was 30 U/mL, and the injected F‐condoliase was 300–360 mU. As shown in Figure 2, the mean area of each substance at 0, 1, and 3 h after injection into the nucleus pulposus was 13.0 ± 4.8, 17.8 ± 10.0, and 18.9 ± 13.7 mm^2^ for F‐condoliase (*n* = 4) and 15.2 ± 1.7, 55.2 ± 16.8, and 108.2 ± 9.6 mm^2^ for fluorescein (*n* = 3). Three hours after injection, the area of fluorescein expanded by approximately 7 times, while that of F‐condoliase only expanded by approximately 1.4 times, showing little diffusion.

### 3.3 Enzyme Activity of Condoliase in Nucleus Pulposus or CS Solution

The condoliase activity in nucleus pulposus or CS solution was evaluated ex vivo and in vitro.

**FIGURE 3:**
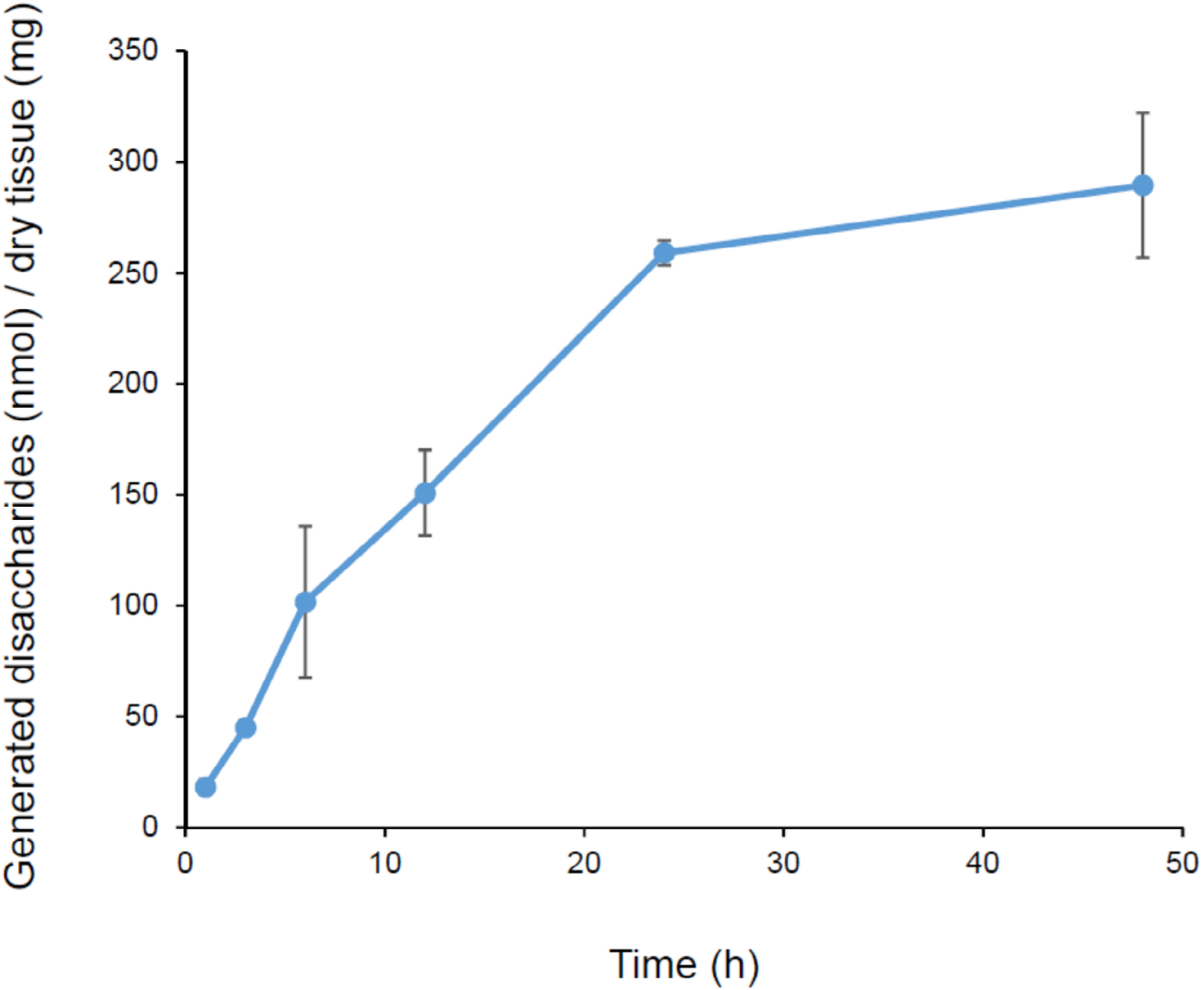
Time course of the release of CS‐disaccharides from porcine nucleus pulposus during condoliase digestion. Excised porcine nucleus pulposus was injected with condoliase (12 μL [15 mU] / 0.1 g excised nucleus pulposus) and incubated at 37°C. The total amount of ΔDi‐0S, ΔDi‐4S, and ΔDi‐6S (generated disaccharides [nmol]) was calculated and divided by the amount of dry tissue (mg). The generated disaccharides (nmol) / dry tissue (mg) at each measurement point are plotted as the mean ± standard deviation (*n* = 3 or 4). The slopes determined by the stepwise linear regression analysis were 10.2 (95% confidence interval [CI]: 7.6 to 12.8; *p* = 0.0011) for 1–24 h and 0.9 (95% CI: −1.7 to 3.7; *p* = 0.3397) for 24–48 h.

First, 15 mU of condoliase was injected per 0.1 g of nucleus pulposus, and the amount of unsaturated disaccharide produced over time was evaluated. After injection, the amounts of CS‐disaccharides significantly increased up to 24 h, and condoliase activity in the nucleus pulposus was maintained for at least 48 h (Figure 3). The slopes determined by the stepwise linear regression analysis with the breakpoint at 24 h were 10.2 (95% confidence interval [CI]: 7.6–12.8; *p* = 0.0011) for 1–24 h and 0.9 (95% CI: −1.7 to 3.7; *p* = 0.3397) for 24–48 h. The total amount of the main CS‐disaccharides, ΔDi‐0S, ΔDi‐4S, and ΔDi‐6S, produced at 48 h was 289 (nmol)/dry tissue (mg). The CS‐disaccharide produced at 48 h accounted for approximately 40% of the CS disaccharide (727 nmol/mg dry tissue) in the nucleus pulposus.

**FIGURE 4:**
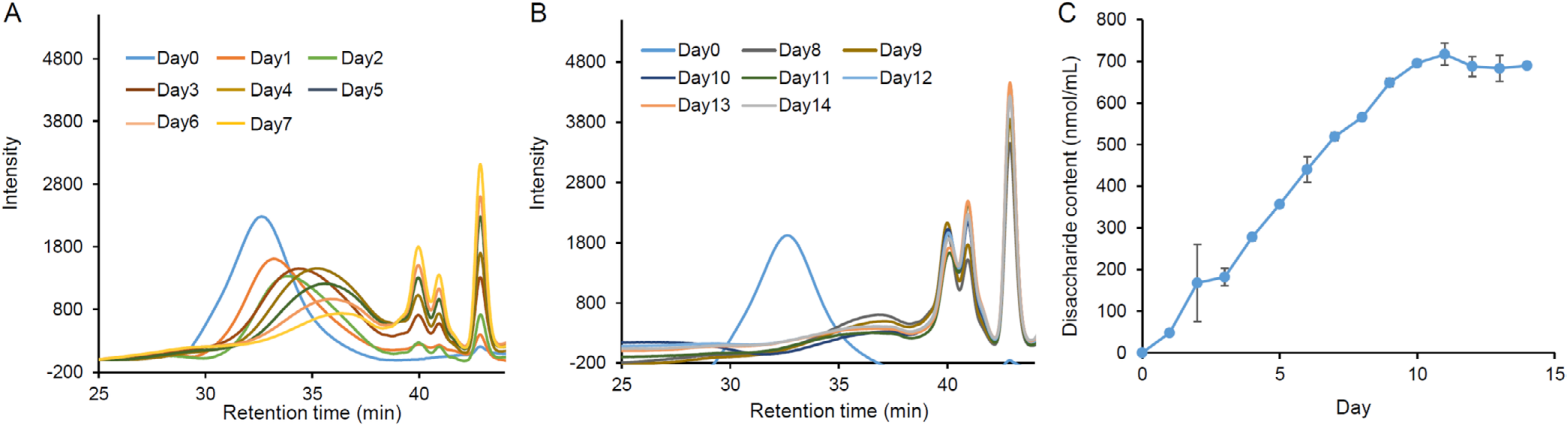
Persistence of the enzymatic activity of condoliase in CS solution. One mL of CSA (1 mg/mL) was mixed with condoliase (0.1 mU) and incubated at 37°C for 14 days. (A) HPSEC profiles of the products of CS digestion from Days 1 to 7. (B) HPSEC profiles of the products of CS digestion from Day 8 to Day 14. (C) Time course of the release of CS‐disaccharides (nmol/mL) from CS during condoliase digestion. The value at each measurement point is plotted as the mean ± standard deviation (*n* = 3). The slopes determined by the stepwise linear regression analysis were 72.5 (95% CI: 68.4 to 76.6; *p* < 0.0001) for the 1st–10th Day and −8.6 (95% CI: −18.7 to 1.4; *p* = 0.0867) for the 10th–14th Day.

Next, we evaluated the persistence of condoliase activity in CS solution (Figure 4). When adding 0.1 mU of enzyme, the CS peak at RT 30–35 min observed before enzyme treatment almost disappeared on Day 7 from the start of the enzyme reaction (Figure 4A,B). In Figure 4C, the slopes determined by the stepwise linear regression analysis with the breakpoint at Day 10 were 72.5 (95% CI: 68.4–76.6; *p* < 0.0001) for 1st–10th Day and −8.6 (95% CI: −18.7 to 1.4; *p* = 0.0867) for 10th–14th Day. This result suggested that most of the CS polymer was digested by condoliase at Day 10, and no undigested CS remained.

### 3.4 Morphology of Nucleus Pulposus With SEM

**FIGURE 5:**
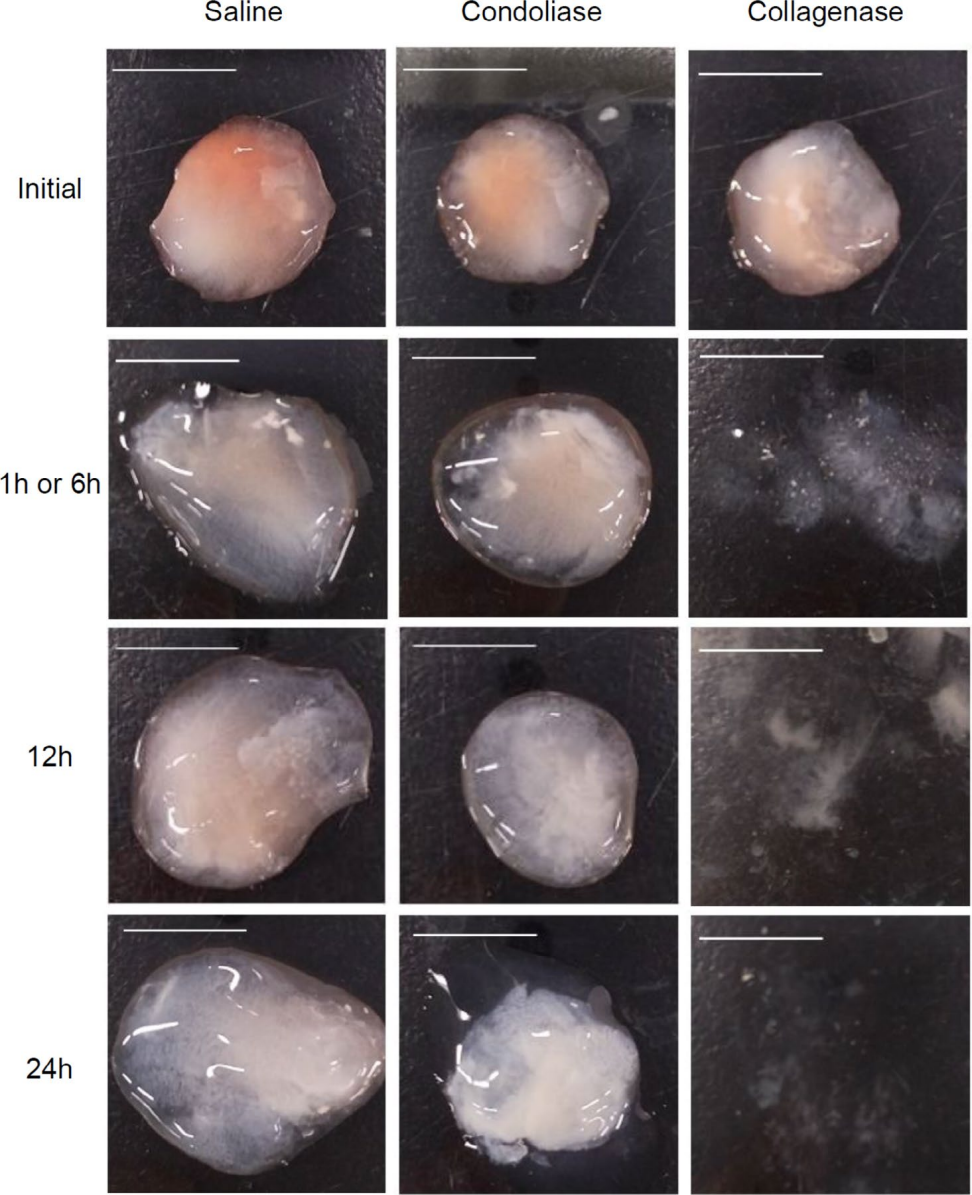
Representative photographic images of porcine nucleus pulposus treated with enzyme. Porcine nucleus pulposus was immersed in 1 mL of each solution: saline, condoliase (1.25 U), and collagenase (1 U). Incubation times at 37°C were 6, 12, and 24 h for saline or condoliase solutions and 1, 12, and 24 h for collagenase solutions. Scale bar = 1 cm.

**FIGURE 6:**
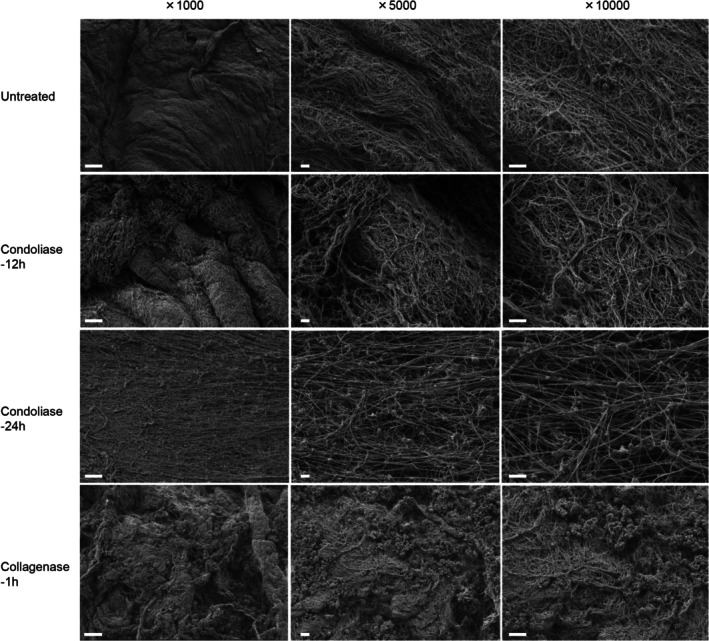
SEM images of porcine nucleus pulposus treated with enzyme. Untreated is a nucleus pulposus that has not been treated with any enzyme or saline. Porcine nucleus pulposus was incubated at 37°C with 1 mL of condoliase (1.25 U) for 12 and 24 h or collagenase (1 U) for 1 h. Scale bar = 10 μm (×1000 magnification). Scale bar = 1 μm (×5000 or ×10 000 magnification). Acceleration voltage: 1 kV.

As shown in Figure 5 and S5, the nucleus pulposus immersed in 1 U/mL collagenase lost its original shape after 1 h of incubation, and after 12 h it liquefied and dispersed. The nucleus pulposus immersed in 1.25 U/mL condoliase maintained its solid morphology even after 24 h of incubation. The nucleus pulposus immersed in saline also maintained its shape. In the SEM images (Figure 6), the fibrous substance observed in the intact nucleus pulposus disappeared after treatment with 1 U/mL collagenase. Therefore, this fibrous substance was suggested to be collagen fibers. The collagen fibers remained in the nucleus pulposus treated with 1.25 U/mL condoliase for 12 or 24 h. Collagen fibers in the nucleus pulposus treated with condoliase for 24 h were separated from each other and became sparse compared to those treated for 12 h.

## 4 Discussion

To examine the behavior of condoliase in the nucleus pulposus, we used isolated porcine nucleus pulposus and obtained new evidence that enhances current understanding of the pharmacological mechanism of condoliase. In this study, condoliase acted on CS in a limited area of the nucleus pulposus rather than all CS contained in the tissue. Condoliase does not degrade collagen fibers; thus, it was difficult to diffuse into the tissue and may have acted on a limited area of CS. Our results explain the possibility that condoliase is a medication with less effect on the structure of the nucleus pulposus.

Aggrecan, the major proteoglycan (PG) contained in the nucleus pulposus, has important IVD functions such as water retention and resistance to compression [14]. Aggrecan, whose MW is approximately 2500 kDa, consists of a large core protein attached to multiple CS (approximately 20 kDa) and keratan sulfate (KS) chains [15]. The MW of CS obtained from animal sources, for example, shark or whale cartilage, is generally more than 15 kDa [13, 16]. The MW of CS from porcine nucleus pulposus in this study was 7.6 kDa, which is smaller than that of CS from other species, except for the CS derived from sturgeon cartilage, which is reported as 8 kDa [16]. However, how the functions of CS in the living body depend on its MW is not clear. Furthermore, the MW of CS in human nucleus pulposus has not been reported. CS in human nucleus pulposus is mostly composed of A‐ and C‐units, with the latter accounting for over 80% of the composition ratio [8]. The porcine nucleus pulposus also contains more than 80% C‐units.

To understand the mechanism of action of condoliase, we evaluated the behavior and enzyme activity of condoliase in the nucleus pulposus. For lumbar discography, it is considered most desirable to position the needle tip in the center of the IVD [17]. According to Okada et al., condoliase should be injected into the center of the IVD to obtain the best outcome [18]. Although the behavior of contrast media inside the nucleus pulposus can be observed under radioscopy, it is not possible to visually detect condoliase. In this study, condoliase was labeled with fluorescein to visually assess the behavior of condoliase in the nucleus pulposus. The F‐condoliase was difficult to diffuse spontaneously and remained close to the injection site in the nucleus pulposus (Figure 2). However, the concentration of F‐condoliase used in this study is higher than that of condoliase used clinically.

Collagen in the nucleus pulposus forms the scaffold structure responsible for tissue rigidity. Aggrecan and hyaluronic acid fill in the gaps between collagen fibers. The network of collagen and aggrecan is hard and difficult to deform. Moreover, aggrecan also offers great resistance to any fluid flow and redistribution of water [15, 19, 20]. As shown in the SEM image (Figure 6), the nucleus pulposus tissue contains a dense matrix of collagen fibers. It was suggested that the size of condoliase (MW 110000, considerably larger than that of fluorescein [MW 332]) impedes its passage through the network structure of collagen fibers. The pharmacological effect of condoliase is exerted through a series of actions beginning with the degradation of CS, the major water‐retaining component in the nucleus pulposus, and ending in the reduction of intradiscal pressure. This study suggested that condoliase remains in the center of the nucleus pulposus, degrades CS, and reduces intradiscal pressure by decreasing water content.

In this study, the amount of condoliase injected into isolated porcine nucleus pulposus was set based on the ratio of nucleus pulposus volume of humans. The ability to maintain the enzymatic activity of condoliase was evaluated based on the quantitative value of the amount of CS disaccharides produced by the enzymatic digestion. As shown in Figure 3, the CS‐disaccharides production nearly reached a plateau value after 24 h. The CS digested by condoliase was a part of the total CS contained in the nucleus pulposus. It was suggested that the enzymatically digested CS in the nucleus pulposus is limited to the area of condoliase retention around the injection site.

As shown in Figure 4, the enzymatic activity of 0.1 mU condoliase was maintained for at least the 10th day in CS solution. On the other hand, condoliase was inactivated within 1 h when stored in saline without CS at 37°C [21]. Therefore, condoliase activity is maintained long term in the presence of undigested CS polymer. If no CS polymer remains in the nucleus pulposus tissue around the injected condoliase, it is possible that the enzyme activity is attenuated or inactivated. In a clinical trial, the time to reach maximum serum concentration of KS after condoliase administration was 1.9 days [4]. Since KS is not a substrate for condoliase, it is possible that KS is transferred into the blood while bound to fragments of PG molecules. These findings also suggested that condoliase administered into the nucleus pulposus significantly degrades CS for at least 24 h.

It was anticipated that condoliase would be a therapeutic drug with less effect on surrounding tissue, including nerves and vessels, because condoliase completely lacks proteolytic activity and acts specifically on the CS of the nucleus pulposus [11, 12]. Using SEM to observe the human nucleus pulposus after the administration of chymopapain, Roggendorf et al. discovered that the collagen network was nearly naked and devoid of ground substance [22]. Using histological staining, Minamisawa et al. ascertained that condoliase acts in human IVD without causing necrosis of chondrocytes and surrounding tissues [6]. This study is the first report to show the morphology of the condoliase‐injected nucleus pulposus using SEM (Figure 6). Collagen fibers that maintain the support structure of the nucleus pulposus were not digested by condoliase. The collagen fiber density of the nucleus pulposus treated with condoliase was sparser after treatment for 24 h than after treatment for 12 h. CS is bound to collagen and other proteins in the living body [23, 24]. We speculated that digestion of CS with condoliase caused the separation between each collagen fiber and the arrangement of fibers to become sparse.

Clinical relevance; Careful consideration of treatment with condoliase injection is required for patients with less degenerated discs, as well as those with severely degenerated discs containing little proteoglycan in the nucleus pulposus, according to Nakajima et al. [25, 26]. However, little is known about the enzymatic behavior of condoliase in the nucleus pulposus regarding diffusibility or enzymatic activity and morphology. The enzymatic properties obtained in this study can be useful metrics to understand and predict the enzymatic behavior or the mechanism of the pharmacological action in the nucleus pulposus during treatment with condoliase. Evidence‐based information may assist surgeons or pain specialists in making a medical decision.

Adams et al. classified into five types of lumbar discogram based on the distribution of injected fluid. The type of discogram is usually associated with the stage of disc degeneration [27]. Various changes occur in the human nucleus pulposus of herniated IVDs, including an increase in type I collagen, a decrease in type II collagen, and a decrease in PG [28, 29]. The Pfirrmann grade differs among patients, and the morphology of the nucleus pulposus changes with the formation of a hernia mass. The behavior of condoliase in the living body may differ depending on the stage of disc degeneration. Furthermore, mechanical loading of intervertebral disc affects the transport of fluids and solutes through the tissue according to Jackson et al. [30].

Human tissue obtained from a lesion by surgery does not provide sufficient material. With human biological specimens, the tissue evaluation may be limited and inadequate. The isolated porcine nucleus pulposus can be a useful tool for pre‐clinical research. However, this study has several limitations. The isolated porcine nucleus pulposus used in this study does not completely mimic the nucleus pulposus in humans, for example, with regard to pathology, disc structure, and pressure. The glycosaminoglycans content and water content are similar in the nucleus pulposus of pigs and humans, respectively, but pig nucleus pulposus had less than half of the collagen in human nucleus pulposus [31, 32]. In addition, pig lumbar disc had higher torsional stiffness than that of human lumbar disc [32]. Further research is required to confirm the homology between species and resolve these issues using methods including histological staining.

In order to use animal‐derived tissues as research tools, it is necessary to understand the differences in their properties between humans and other animals. Based on the findings, we would like to elucidate the morphology of human nucleus pulposus treated with condoliase, or the relationship between enzymatic behavior and therapeutic efficacy in the future.

## 5 Conclusion

We provide useful information for a greater understanding of the behavior and pharmacological effects of condoliase in the isolated porcine nucleus pulposus. These results can also be useful metrics when evaluating biological samples of condoliase‐treated human tissue in the future.

## Author Contributions

**Ippei Watanabe:** designed the experiments, performed the experiments, data curation, data interpretation, and writing – original draft preparation; **Ko Takeda:** investigation, data interpretation, writing – reviewing and editing; **Taiichi Shirogane:** project administration, supervision, and reviewing the manuscript from clinical and scientific standpoints. All authors have read and approved the final version of the manuscript.

## Conflicts of Interest

Ippei Watanabe, Ko Takeda, Taiichi Shirogane are employees of Seikagaku Corp.

## Supporting information


**Figure S1:** The area of F‐condoliase or fluorescein injected in the nucleus pulposus. Images are opened in the ImageJ software and the areas are measured. “16‐bit” is selected in the “Type” popup window, and the original image is then automatically converted to grayscale. To set the threshold for the fluorescence signal, the threshold values used for binarization are as follows: lower threshold: 23, upper threshold: 100. As shown in the above figure, the area of the fluorescent signal was calculated by automatic selection using the “wand tool” of ImageJ. When the fluorescent signals could not be grouped together using the “wand tool,” they were grouped separately and the sum of each was calculated.
**Figure S2:** HPSEC profiles of CSC and CSC treated with actinase. (A) Blue line; 2 mg/mL CSC (in Tris–HCl buffer), Orange line; CSC was treated with 2% actinase (in Tris–HCl buffer pH 8.0), and the mixture was incubated at 55°C for overnight. After incubation, the mixture was heated to 100°C for 10 min. (B) 2% Actinase (in Tris–HCl buffer pH 8.0).
**Figure S3:**. Absorption spectrum of F‐condoliase. Absorbance at 250–600 nm of F‐cABC was measured using a spectrophotometer (UV‐1900i, Shimadzu, Kyoto). The stock solution was diluted 10 (gray), 15 (orange), and 20 (blue) times with distilled water. The absorbances of F‐cABC at 280 nm and 500 nm were as follows: 0.473 and 0.297 for the 10‐fold diluted solution, 0.314 and 0.188 for the 15‐fold diluted solution, and 0.227 and 0.137 for the 20‐fold diluted solution. The ration 280 nm/500 nm of F‐condoliase was 1.6 ± 0.04, and the labeling rate was 3.5 ± 0.1 (*n* = 3).
**Figure S4:**. Time course of reactivity of F‐condoliase to CSC. A 1 mL of CSC (1 mg/mL) was mixed with 10 μL of F‐condoliase and incubated at pH 7.0 and 37°C (*n* = 3).
**Figure S5:** Photographic images of porcine nucleus pulposus treated with enzyme. Porcine nucleus pulposus was immersed in 1 mL of each solution; saline, condoliase (1.25 U), and collagenase (1 U). Incubation times at 37°C were 6, 12, and 24 h for saline or condoliase solutions, and 1, 12, and 24 h for collagenase solutions. Scale bar = 1 cm.
