# Supplementary material for: Enzymatic Behavior of Condoliase in Porcine Nucleus Pulposus: Ex Vivo and In Vitro Assessment
Source: JOR Spine. 2025 Oct 19;8(4):e70131. doi: 10.1002/jsp2.70131 (PMC12535817; doi:10.1002/jsp2.70131)
Supplement: Supplementary file 1 — Figure S1: The area of F‐condoliase or fluorescein injected in the nucleus pulposus. Images are opened in the ImageJ software and the areas are measured. “16‐bit” is selected in the “Type” popup window, and the original image is then automatically converted to grayscale. To set the threshold for the fluorescence signal, the threshold values used for binarization are as follows: lower threshold: 23, upper threshold: 100. As shown in the above figure, the area of the fluorescent signal was calculated by automatic selection using the “wand tool” of ImageJ. When the fluorescent signals could not be grouped together using the “wand tool,” they were grouped separately and the sum of each was calculated. Figure S2: HPSEC profiles of CSC and CSC treated with actinase. (A) Blue line; 2 mg/mL CSC (in Tris–HCl buffer), Orange line; CSC was treated with 2% actinase (in Tris–HCl buffer pH 8.0), and the mixture was incubated at 55°C for overnight. After incubation, the mixture was heated to 100°C for 10 min. (B) 2% Actinase (in Tris–HCl buffer pH 8.0). Figure S3:. Absorption spectrum of F‐condoliase. Absorbance at 250–600 nm of F‐cABC was measured using a spectrophotometer (UV‐1900i, Shimadzu, Kyoto). The stock solution was diluted 10 (gray), 15 (orange), and 20 (blue) times with distilled water. The absorbances of F‐cABC at 280 nm and 500 nm were as follows: 0.473 and 0.297 for the 10‐fold diluted solution, 0.314 and 0.188 for the 15‐fold diluted solution, and 0.227 and 0.137 for the 20‐fold diluted solution. The ration 280 nm/500 nm of F‐condoliase was 1.6 ± 0.04, and the labeling rate was 3.5 ± 0.1 (n = 3). Figure S4:. Time course of reactivity of F‐condoliase to CSC. A 1 mL of CSC (1 mg/mL) was mixed with 10 μL of F‐condoliase and incubated at pH 7.0 and 37°C (n = 3). Figure S5: Photographic images of porcine nucleus pulposus treated with enzyme. Porcine nucleus pulposus was immersed in 1 mL of each solution; saline, condoliase (1.25 U), and collagenase (1 U). Incubation time [file JSP2-8-e70131-s001.docx]

**Supplementary material**


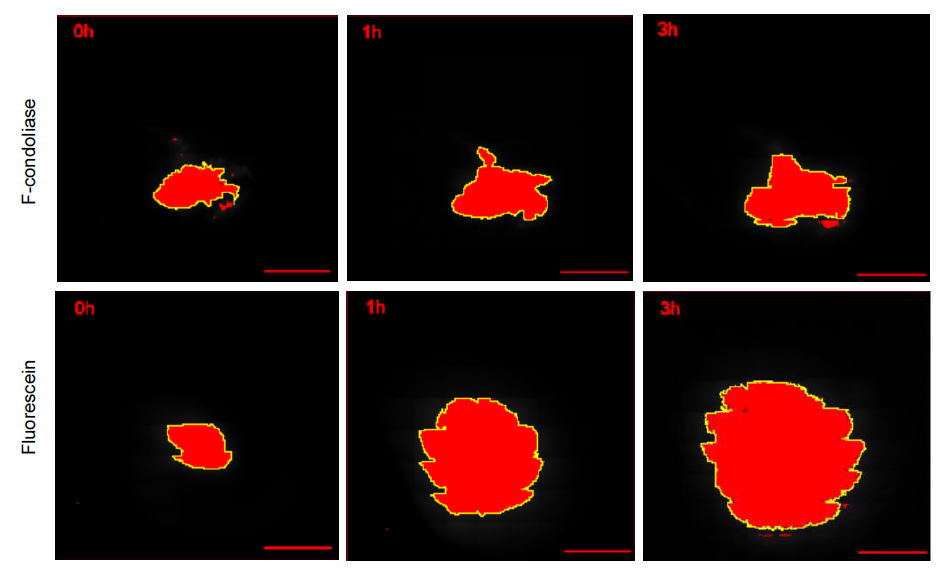


**Figure S1. The area of F-condoliase or fluorescein injected in the nucleus pulposus.** Images are opened in the ImageJ software and the areas are measured. “16-bit” is selected in the “Type” popup window, and the original image is then automatically converted to grayscale. To set the threshold for the fluorescence signal, the threshold values used for binarization are as follows: lower threshold: 23, upper threshold: 100. As shown in the above figure, the area of ​​the fluorescent signal was calculated by automatic selection using the “wand tool” of ImageJ. When the fluorescent signals could not be grouped together using the "wand tool," they were grouped separately and the sum of each was calculated.


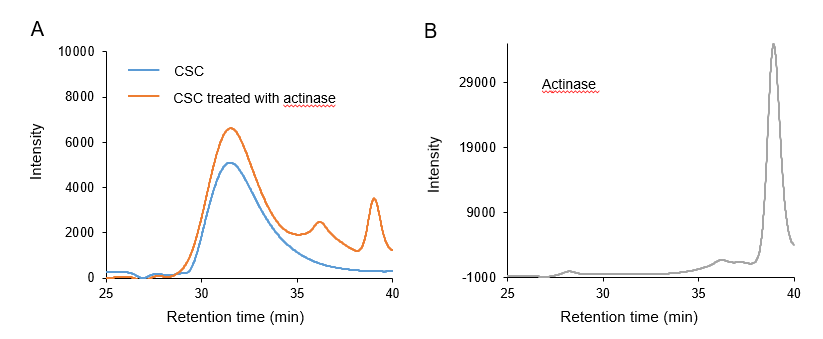


**Figure S2. HPSEC profiles of CSC and CSC treated with actinase**. (A) Blue line; 2 mg/mL CSC (in Tris-HCl buffer), Orange line; CSC was treated with 2% actinase (in Tris-HCl buffer pH8.0), and the mixture was incubated at 55 °C for overnight. After incubation, the mixture was heated to 100 °C for 10 min. (B) 2% Actinase (in Tris-HCl buffer pH8.0).

**
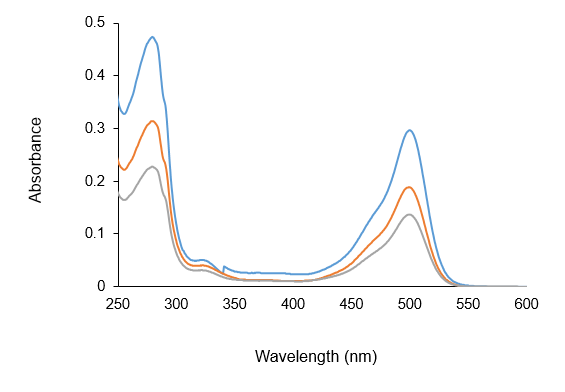
**

**Figure S3**. **Absorption spectrum of F-condoliase.** Absorbance at 250-600 nm of F-cABC was measured using a spectrophotometer (UV-1900i, Shimadzu, Kyoto). The stock solution was diluted 10 (gray), 15 (orange), and 20 (blue) times with distilled water. The absorbances of F-cABC at 280 nm and 500 nm were as follows: 0.473 and 0.297 for the 10-fold diluted solution, 0.314 and 0.188 for the 15-fold diluted solution, and 0.227 and 0.137 for the 20-fold diluted solution. The ration 280 nm / 500 nm of F-condoliase was 1.6 ± 0.04, and the labeling rate was 3.5 ± 0.1 (n = 3).


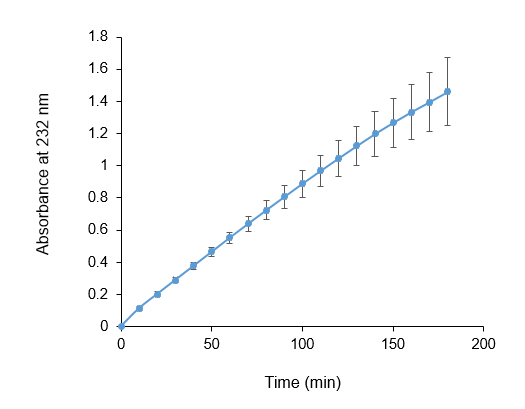


**Figure S4**. **Time course of reactivity of F-condoliase to CSC.** A 1 mL of CSC (1 mg/mL) was mixed with 10 µL of F-condoliase and incubated at pH 7.0 and 37 °C (n = 3).


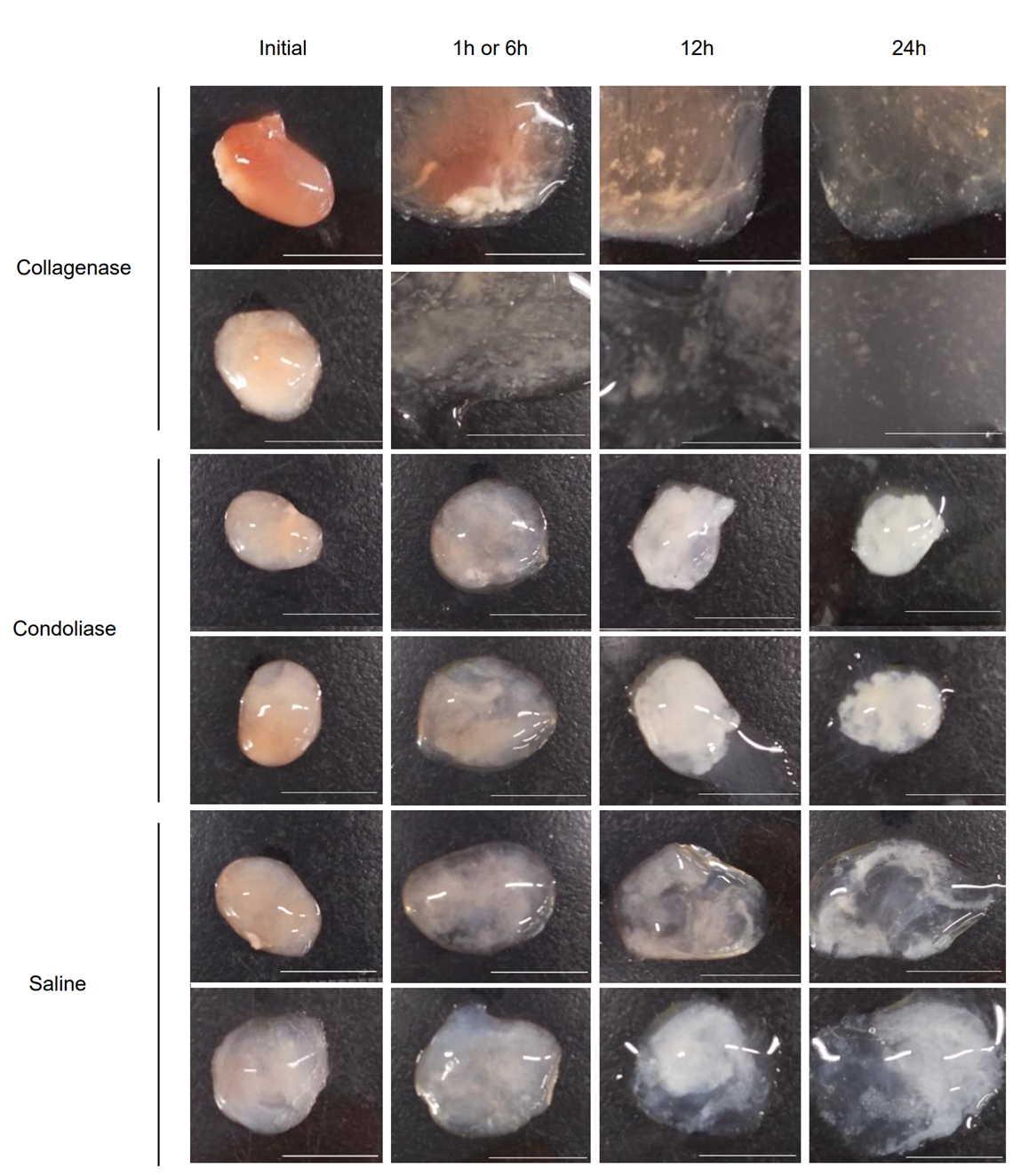


**Fig. S5 Photographic images of porcine nucleus pulposus treated with enzyme.** Porcine nucleus pulposus was immersed in 1 mL of each solution; saline, condoliase (1.25 U), and collagenase (1 U). Incubation times at 37°C were 6, 12, and 24 h for saline or condoliase solutions, and 1, 12, and 24 h for collagenase solutions. Scale bar = 1 cm.
